# Beliefs in Conspiracy Theories and Misinformation About COVID-19: Comparative Perspectives on the Role of Anxiety, Depression and Exposure to and Trust in Information Sources

**DOI:** 10.3389/fpsyg.2021.646394

**Published:** 2021-04-16

**Authors:** David De Coninck, Thomas Frissen, Koen Matthijs, Leen d’Haenens, Grégoire Lits, Olivier Champagne-Poirier, Marie-Eve Carignan, Marc D. David, Nathalie Pignard-Cheynel, Sébastien Salerno, Melissa Généreux

**Affiliations:** ^1^Centre for Sociological Research, KU Leuven, Leuven, Belgium; ^2^Department of Technology and Society Studies, Faculty of Arts and Social Sciences, Maastricht University, Maastricht, Netherlands; ^3^Institute for Media Studies, KU Leuven, Leuven, Belgium; ^4^Institut Langage et Communication, Université catholique de Louvain, Louvain-la-Neuve, Belgium; ^5^Département de Communication, Faculté des Lettres et Sciences Humaines, Université de Sherbrooke, Sherbrooke, QC, Canada; ^6^Université de Neuchâtel, Académie du Journalisme et des Médias, Neuchâtel, Switzerland; ^7^Medi@Lab, Université de Genève, Genève, Switzerland; ^8^Department of Community Health Sciences, Faculty of Medicine and Health Sciences, Université de Sherbrooke, Sherbrooke, QC, Canada

**Keywords:** COVID-19, conspiracy beliefs, misinformation beliefs, information sources, pandemic, conspiracy theories

## Abstract

While COVID-19 spreads aggressively and rapidly across the globe, many societies have also witnessed the spread of other viral phenomena like misinformation, conspiracy theories, and general mass suspicions about what is really going on. This study investigates how exposure to and trust in information sources, and anxiety and depression, are associated with conspiracy and misinformation beliefs in eight countries/regions (Belgium, Canada, England, Philippines, Hong Kong, New Zealand, United States, Switzerland) during the COVID-19 pandemic. Data were collected in an online survey fielded from May 29, 2020 to June 12, 2020, resulting in a multinational representative sample of 8,806 adult respondents. Results indicate that greater exposure to traditional media (television, radio, newspapers) is associated with lower conspiracy and misinformation beliefs, while exposure to politicians and digital media and personal contacts are associated with greater conspiracy and misinformation beliefs. Exposure to health experts is associated with lower conspiracy beliefs only. Higher feelings of depression are also associated with greater conspiracy and misinformation beliefs. We also found relevant group- and country differences. We discuss the implications of these results.

## Introduction

While the SARS-CoV-2 virus—responsible for causing the COVID-19 disease—spreads aggressively and rapidly across the globe, many societies have also witnessed the spread of other seemingly viral phenomena such as fake news, conspiracy theories, and general mass suspicions about what is really going on. Some of the most prevailing narratives are the ones claiming that the virus is caused by 5G cellular technology (Vincent, 2020) or that Bill Gates uses the virus to enslave humanity by enforcing a global vaccination and surveillance program (Shahsavari et al., 2020). Even though most of these stories were quickly debunked and proven untrue, the pervasiveness of misinformation and conspiracy theories on social media and in the news cycle has led the Director-General of the World Health Organization (WHO) to warn that “*We’re not just fighting an epidemic; we’re fighting an infodemic. Fake news spreads faster and more easily than this virus, and is just as dangerous*” (WHO, 2020a).

The spread of false and/or misleading information is not new. A brief peak into the twentieth century provides us with examples such as Joseph Goebbels’s machinery of *Public Enlightenment*. However, today’s information ecosystem has drastically changed the ways in which mis- and disinformation are produced, disseminated, and consumed (Benkler et al., 2018; Törnberg, 2018). Social media platforms and digital technologies have facilitated high-speed information sharing between news media producers and consumers, as well as cross-platform information cascades (Shu et al., 2016; Vosoughi et al., 2018). Within these online environments, false and fake narratives tend to outperform real news in terms of popularity and audience engagements (Silverman, 2016). As a result, narratives of conspiracy theories and misinformation spread quickly (Venturini, 2019; Gallotti et al., 2020; Garfin et al., 2020). This is especially the case in times of societal crises such as the COVID-19 pandemic (van Prooijen and Douglas, 2017; De Coninck et al., 2020; Imhoff and Lamberty, 2020; Knuutila et al., 2020), as rumors, conspiracy theories, and “alternative truths” tend to thrive in environments of high fear, low confidence, and low trust (Shahsavari et al., 2020). There is a rich body of literature discussing what exactly constitutes “fake news” (Farkas and Schou, 2018; Tandoc et al., 2018) or “misinformation” (Benkler et al., 2018). It is beyond the scope of the current study to review this literature. We consider misinformation (or fake news) as “*publishing wrong information without meaning to be wrong or having a political purpose in communicating false information*,” and disinformation (or conspiracy theories) as *‘manipulating and misleading people intentionally to achieve political ends’* (Benkler et al., 2018, p. 24). More specifically, disinformation and conspiracy theories “are attempts to explain the ultimate causes of significant social and political events and circumstances with claims of secret plots by two or more powerful actors” (Douglas et al., 2019, p. 4).

While some hold the belief that misinformation and conspiracy theories are fringe phenomena or mundane (digital) artifacts with small impact on real-world actions, several events during the COVID-19 pandemic across different countries demonstrate the opposite. For example, in reaction to the conspiracy theories that claim that 5G cellular network is the cause of the disease^1^, over 200 incidents have been reported of attacks against telecom workers in the U.K. (Vincent, 2020), and numerous mobile telecom masts were set on fire in the Netherlands (Wassens, 2020). Furthermore, previous studies have shown that exposure to disease-related conspiracy theories is associated with lower vaccination intentions (Jolley and Douglas, 2014), lower levels of trust in governmental and health institutions (Lutkenhaus et al., 2019), and less willingness to follow restrictive measures to curtail further propagation of the disease (Imhoff and Lamberty, 2020). Evidence from England also shows that COVID-19-related conspiracy thinking is associated with less adherence to all government guidelines and less willingness to take diagnostic or antibody tests or to be vaccinated (Freeman et al., 2020). To highlight the potentially far-reaching and damaging effects of mis- and disinformation, Saiful Islam et al. (2020) estimate that widespread misinformation on social media on the consumption of highly concentrated alcohol that could disinfect the body and kill the coronavirus, resulted in approximately 800 deaths and 5,800 hospitalizations worldwide. It is therefore argued that the COVID-19 crisis is one of the first deeply mediatized global pandemics (Hepp, 2020), following earlier bird flu and Ebola epidemics (Joffe, 2011).

Previous findings show that conspiracy thinking is associated with an avoidance of established and traditional media (television, radio, newspapers) and with a tendency to acquire information mainly through digital media, including the internet and social media (Vosoughi et al., 2018; Boberg et al., 2020; Humprecht et al., 2020). The digital media ecosystem—with its socially networked architecture, trolls, and automated bots (Zannettou et al., 2018)—rather than the traditional news media, has been considered a hotbed for mis- and disinformation, such as conspiracy theories (Shu et al., 2016; Vosoughi et al., 2018). In line with this literature, we expect that exposure to digital media will be associated with greater conspiracy (H1a) and misinformation (H1b) beliefs. Exposure to traditional media, which regularly undertake efforts to debunk conspiracy theories and misinformation (Hollander, 2017), is expected to be associated with lower conspiracy (H2a) and misinformation (H2b) beliefs.

Aside from effects of mere exposure, trust in these media are also expected to play a role. Research has shown that distrust in traditional news media leads to selective exposure to news (Swire et al., 2017) and increases the use of alternative sources, such as digital media that distribute disinformation (Boberg et al., 2020). In other words, in environments in which distrust in traditional news media is higher, people are less likely to be exposed to different sources of political information and to critically evaluate these sources (Benkler et al., 2018; Humprecht et al., 2020). Based on this reasoning, it can be assumed that resilience to conspiracies and misinformation is lower in societies where distrust in professional news media is high. Thus, we expect that the effect of exposure to information sources on conspiracy and misinformation beliefs is moderated by trust in these sources (H3).

Self-evidently, in times of a global health emergency, such as the COVID-19 pandemic with its rapid spread and the high mortality rate, people are confronted with a monumental state of uncertainty and threat. In numerous recent studies it has been demonstrated that this continuous and unprecedented sense of uncertainty is inevitably related to increased levels of stress and psychological distress (Barzilay et al., 2020; Salari et al., 2020). Recent Chinese data have shown that during the COVID-19 pandemic 34.13% of the people experienced moderate to severe stress symptoms (Qiu et al., 2020). Furthermore, the typical stress levels associated with the pandemic have even appropriated the introduction of a new syndrome called “COVID stress syndrome” (Taylor et al., 2020), which has been consistently found to be linked to feelings of depression and anxiety in the general population (Barzilay et al., 2020; Salari et al., 2020). That elevated levels of (sudden) stress activate feelings or symptoms of depression is a well-documented process in the psychological literature. In order alleviate the feelings of stress and to regain a sense of control of the situation in which people find themselves today, one could experience the need to cognitively project personal feelings of threat and stress to a social out-group or power (Poon et al., 2020). This is where narratives and the sense-making function of conspiracy theories come into play. Although sense-making mechanisms (e.g., obtaining information from different types of sources to make sense of the COVID-situation) are intended to reduce anxious or depressive feelings, they often actually result in a higher susceptibility to conspiracy beliefs (van Prooijen and Douglas, 2017; van Prooijen, 2017; Šrol et al., 2021). Conspiracy beliefs are then a “*feature of the mind”* that help shaping certainty and control in times of uncertainty and stress (Kossowska and Bukowski, 2015; Moulding et al., 2016), which makes people with depressogenic schemata extra susceptible for this “feature.” Furthermore, cognitive theoretical models have suggested that negative schemata also catalyze a need for more information about the stressful situation in order to make the threat more predictable or controllable. Yet, recent studies have found that seeking for information actually backfires and could even exacerbate levels of stress because of the fact that one encounters new, stress-evoking information such as graphic imagery in mainstream news media, but also misinformation and conspiracy theories (Taylor et al., 2020). Based on this literature, we expect that feelings of anxiety (H4) and depression (H5) mediate the positive association between exposure to information sources and conspiracy and misinformation beliefs.

### The Present Study

The overarching goal of this international study was to better understand how information is delivered and communicated by authorities and media in the context of the COVID-19 pandemic, and how it is received, understood, and used by the public in eight countries/regions: Belgium, Canada, England, Hong Kong, New Zealand, the Philippines, Switzerland, and the United States. The selection of these countries/regions was informed by Humprecht et al.’s (2020) framework for cross-national comparative research on disinformation. Based on several indicators (e.g., populism, polarization, media trust, social media use, strength of public broadcaster), they develop clusters of countries to inform cross-national research on disinformation. They find that most Western and Central European countries (including Belgium and Switzerland) belong to a single cluster, with a media-supportive and consensual political system. Despite some differences in media systems, their analysis finds that the United Kingdom and Canada also belong to this cluster. These countries “seem to be well equipped to face the challenges of the digital information age because they have stable, trusted institutions that enable citizens to obtain independent information and uncover manipulation attempts” (Humprecht et al., 2020, p. 507). In their study, the United States is a unique case. It does not belong to any cluster, given its polarized political and media environment, which has created a fertile ground for the spread of disinformation today. Political communication in the United States is characterized by populist rhetoric, while media coverage has become more partisan and, as a consequence, trust in the media has decreased (Humprecht et al., 2020). Although not included in the current framework, we expect that the Philippines [with the election of president Rodrigo Duterte (Webb and Curato, 2019)] and Hong Kong [with its highly partisan media landscape and the on-going polarization around the question of independence (Wu and Shen, 2020)] share several characteristics with the U.S., warranting their selection. New Zealand is the only country which does not clearly fit into this disinformation framework, but this country was mainly selected for its approach to the COVID-19-pandemic. At the time of the study, nearly all countries worldwide were still combating the pandemic, while New Zealand—thanks to a highly restrictive approach early on—had effectively eliminated COVID-19 within its borders (Cousins, 2020). While we cannot make predictors for all countries, based on this literature we expect that conspiracy and misinformation beliefs are low in countries with a media-supportive and consensual political system (H6a), but high in countries with a polarized political and media environment (H6b).

## Data and Measures

### Design

We collected data through online surveys among a sample of the adult population in eight countries/regions: Belgium, Canada, England, Hong Kong, New Zealand, the Philippines, Switzerland, and the United States (*N* = 8,806). The construction of the online survey was based on the Knowledge–Attitude–Practice model (Bettinghaus, 1986) and, therefore, explored a wide range of aspects, going from risk perceptions and beliefs to positive/negative attitudes and adaptive/maladaptive behaviors. Sociodemographic characteristics were also assessed. The survey contained closed-ended questions only and lasted an average of 18 min per participant. It was pretested among 600 Canadian adults from April 8, 2020 to April 11, 2020, and validated in five different languages (i.e., English, Dutch, Filipino, French, German, Italian, and Chinese). The final surveys were fielded from May 29, 2020 to June 12, 2020 in all countries/regions. This study was approved by the Research Ethics Board of the CIUSSS de l’Estrie—CHUS (HEC ref: 2020-3674).

### Selection of Participants

Recruitment and data collection were carried out by only two polling firms, with the collaboration of international partners, to ensure the standardization of the whole process. Any adults (≥ 18 years) living in each of the eight countries/regions listed above and able to answer an online questionnaire were eligible to participate in the online survey. Participants were randomly recruited from online panels. Several sources were used for the recruitment of panel members, including (a) random recruitment using traditional and mobile telephone methodologies, i.e., recruitment through the firm’s call center, and (b) recruitment by invitation, through social media (Facebook and Instagram), through offline recruitment, and through partner programs and campaigns such as the friend recommendation program. Significant efforts were made to maximize the representativeness of the sample by using software generating representative samples of the population and by including hard-to-reach groups through targeted recruitment. The final sample was composed of approximately 1,000 adults per country/region (Généreux et al., 2020). See Supplementary Appendix A for a comparison of our study sample to the population in the different countries under study in terms of age and household composition.

### Measures

#### Belief in Conspiracy Theories and Misinformation

We developed two indices regarding *belief in conspiracy theories* (e.g., the pharmaceutical industry is involved in the spread of the coronavirus), one with three items (presented in all regions) and another one with six items (presented in all regions except Hong Kong), each presenting possible conspiracy theories regarding the coronavirus disease. The items originated from a Pew Research Center and Fondation Jean-Jaurès/Conspiracy Watch survey, which was one of the only sources available about COVID-19 and conspiracy beliefs when this study was developed (Fondation Jean Jaurès, 2020). Answer options ranged from 1 (do not agree at all) to 10 (fully agree). Principal component analysis indicated a single component with high internal consistency for both scales (three-item α = 0.77; six-item α = 0.86). For the exact wording of items and more information regarding the scales, see Supplementary Appendix B.

*Belief in misinformation* was measured through five items, each presenting a news item regarding the coronavirus which was untrue (but not linked to conspiracies) (e.g., the coronavirus cannot be transmitted in warm countries). These items originated from the WHO Mythbusters, a digital platform developed by the WHO to combat misinformation and fake news regarding a number of topics (WHO, 2020b). Answer options ranged from 1 (do not agree at all) to 10 (fully agree). Principal component analysis on these five items indicated a single component with high internal consistency (α = 0.86). These factor scores were saved and used in subsequent analyses. For the exact wording of each item and more information regarding the scale, see Supplementary Appendix C.

#### COVID-19 Information Sources

Twelve items were used to assess which channels were used by respondents to gather information about the new coronavirus: federal government, local government, politicians, WHO, health professionals in the media, public health authorities (via press conferences), television, radio, newspapers (on- and offline), social media, the internet, and friends/family. For each mode of information, answer options ranged from 1 (never) to 4 (mainly/always). Principal component analysis on these items indicated four components with an Eigenvalue > 1 and with moderate to high internal consistency. These components were: information through public health experts (α = 0.70), political actors (α = 0.67), traditional media (α = 0.71), digital media and personal contacts (α = 0.73). In the descriptive analyses, mean scores of these components were used for ease of interpretation, while factor scores were used in the SEM to increase model parsimony.

#### Trust in COVID-19 Information Sources

Seven items were used to assess trust in different actors and information sources within society: scientists, doctors and health experts, national health organizations, global health organizations, news organizations, government, politicians, people you know. In order to remain in line with the sources of information, we calculated the mean score of the three items regarding health actors (scientists, doctors and health experts, national health organizations, global health organizations) and the mean score of the two items regarding political actors (government, politicians). Answer options ranged from 1 (do not trust at all) to 10 (fully trust). In the descriptive analyses, mean scores of these components were used for ease of interpretation, while factor scores were used in the SEM to increase model parsimony.

#### Anxiety and Depression

Two psychological states were assessed: generalized anxiety disorder (GAD) and major depression episode (MDE), using the GAD-7 (Swinson, 2006) and the Patient Health Questionnaire-9 (PHQ-9) scales (Levis et al., 2019), respectively. These two scales are based on the diagnostic criteria for GAD and MDE described in DSM-IV. These seven and nine item scales, respectively, are primarily designed for use by health professionals but are also regularly used in population-based studies. Answer options ranged from 0 to 3, with the high end indicating greater anxiety or depression. We calculated the aggregate score of the items in each scale to use in subsequent analyses.

#### Socio-Demographic Characteristics

Respondents were asked to indicate age, which was categorized for the purpose of the ANOVA (Table 4). Categories were 18–34, 35–54, 55+. Gender was measured by four options (1 = male, 2 = female, 3 = other, 4 = prefer not to answer). Due to the small group size, those identifying as other (*n* = 18) and those who preferred not to answer (*n* = 6) were indicated as missing. Information regarding educational attainment was adapted for each country and harmonized following the data collection (1 = secondary education or lower, 2 = tertiary education or higher) (Table 1). An overview of the Pearson correlations can be found in Table 2.

**TABLE 1:** Descriptive results of individual-level variables (in% or mean scores).

	Belgium	Canada	England	Hong Kong	New Zealand	Philippines	United States	Switzerland	Total
**Age (mean)**	48.9	48.0	47.5	46.3	46.6	38.2	47.8	49.3	46.6
**Gender (%)**									
Male	49	48	49	45	48	49	49	48	48
Female	51	51	51	55	51	50	51	52	52
**Educational attainment (%)**									
Secondary education or lower	65	32	60	38	34	41	24	44	49
Tertiary education or higher	35	68	39	61	64	57	76	55	51
**Information sources (mean)**									
Health experts	2.4	2.5	2.5	2.5	2.5	3.1	2.5	2.4	2.6
Political actors	2.1	2.4	2.3	2.1	2.3	2.6	2.2	2.4	2.3
Traditional media	2.5	2.1	2.2	2.5	2.4	2.9	2.2	2.4	2.4
Digital media and personal contacts	1.7	1.9	2.0	2.4	2.0	2.8	2.1	2.0	2.1
**Trust in information sources (mean)**									
Health experts	6.9	7.6	7.5	6.7	7.6	7.8	7.1	7.1	7.3
Political actors	4.7	6.2	5.5	5.0	6.8	6.5	4.9	6.4	5.8
Traditional media	5.8	6.3	6.0	6.5	6.3	7.1	6.0	6.1	6.3
Personal contacts	7.6	7.7	7.7	7.2	8.1	7.6	7.6	7.7	7.6
**GAD** (mean)	4.9	5.6	6.2	6.4	5.0	6.4	6.8	4.0	5.7
**PHQ** (mean)	5.0	6.4	7.4	7.0	6.3	6.9	7.6	5.1	6.4
***N***	1,015	1,501	1,041	1,140	1,000	1,041	1,065	1,003	8,806

*1% of respondents are missing for gender, and 1.5% for educational attainment.*

**TABLE 2:** Pearson correlations, mean scores, and standard deviations of the study variables.

	Mean	SD	1	2	3	4	5	6	7	8	9	10	11	12
1. Misinformation beliefs	3.44	2.04	1	0.60**	0.08**	0.18**	0.14**	0.37**	0.01	0.22**	0.16**	0.09**	0.20**	0.24**
2. Conspiracy beliefs	4.66	2.27		1	−0.03*	0.01	0.05**	0.33**	−0.19**	−0.08**	−0.01	−0.16**	0.23**	0.26**
3. Exposure: Health experts	2.55	0.74			1	0.54**	0.44**	0.29**	0.47**	0.30**	0.34**	0.38**	0.05**	0.02
4. Exposure: Political actors	2.31	0.75				1	0.40**	0.26**	0.36**	0.50**	0.29**	0.42**	0.03**	0.04**
5. Exposure: Traditional media	2.39	0.78					1	0.41**	0.26**	0.26**	0.38**	0.25**	0.03**	0.01
6. Exposure: Digital media and personal contacts	2.12	0.75						1	0.05**	0.12**	0.18**	0.06**	0.20**	0.19**
7. Trust: Health experts	7.29	1.87							1	0.61**	0.56**	0.81**	−0.06**	−0.06**
8. Trust: Political actors	5.77	2.45								1	0.52**	0.80**	−0.07**	−0.05**
9. Trust: Traditional media	6.26	2.24									1	0.54**	−0.01	0.00
10. Trust: Personal contacts	7.64	1.66										1	−0.07**	−0.06**
11. GAD	5.67	5.48											1	0.82**
12. PHQ	6.43	6.41												1

*p < 0.05; **p < 0.01.

### Analytic Plan

As mentioned above, we developed two measures regarding belief in conspiracy theories; one with three items and another with six items. In this analysis, we present the results of the analyses per country using the three-item conspiracy scale because the additional items were not presented in Hong Kong. We conducted robustness analyses with all countries combined (Supplementary Appendix E) and with the six-item scale (see Supplementary Appendix F) and found no notable differences with the results based on the three-item scale.

In order to investigate country and sociodemographic differences in conspiracy theory and misinformation beliefs, we used independent samples *t*-tests and one-way ANOVA tests. We then estimated a structural equation model (SEM) for each country or region to investigate associations of exposure to information sources with anxiety and depression, and associations of exposure to and trust in information sources with conspiracy and misinformation beliefs. We also investigated if and how trust in information moderated the effect of exposure. In this model, we controlled for socio-demographic characteristics. We estimated a SEM because of its advantages over OLS regression in three ways in the current study. First, SEM allows for the incorporation of measurement error and offers greater power to detect effects, which is even more important for interaction terms (which we will include in our model) (Sardeshmukh and Vandenberg, 2017). Second, it can test all mediated effects simultaneously if there are multiple mediators—as is the case here. In this study, the relationship between exposure to information sources and conspiracy/misinformation beliefs may be mediated by both anxiety and depression. The SEM analysis allows the specification of these relationships when testing the joint mediating effects of anxiety and depression. SEM can also compare different mediated effects to determine which one is the largest or test if a specific mediated effect is larger than the direct effect (Li, 2011). Third, SEM remains the preferred method for a confirmatory rather than exploratory approach, i.e., for hypothesis testing and multivariate analyses of structural theory (Lei and Wu, 2007; Frissen, 2021). In that sense, a SEM is desired if we wish to determine to what extent collected data are consistent with specific hypotheses (as is the case here). Hence, in the current study, we chose for SEM as it proves to be a robust way to test whether the expectations as discussed above are confirmed by the data from large-scale samples of eight COVID-affected countries from multiple regions in the world.

## Results

In terms of belief in conspiracy theories, one-way ANOVA results signaled significant differences between countries (Table 3). Mean scores indicated that respondents from the Philippines (*M* = 5.83), the United States (*M* = 5.19), and Hong Kong (*M* = 5.03) reported the highest scores with regards to conspiracy beliefs. Respondents from Switzerland (*M* = 4.31), but especially Canada (*M* = 3.95) and New Zealand (*M* = 3.86) reported the lowest scores. As for misinformation beliefs, results again pointed to significant country differences. Respondents from same three countries [Philippines (*M* = 4.91), Hong Kong (*M* = 4.06), United States (*M* = 3.73)] reported the highest belief in misinformation, while respondents from New Zealand (*M* = 3.05), Canada (*M* = 2.75), and Belgium (*M* = 2.62) reported the lowest beliefs in misinformation. These results support the assumption in H6a: respondents from countries with a media-supportive and consensual political system in this study (Belgium, Switzerland, Canada, England) report some of the lowest conspiracy/misinformation beliefs, although scores for English respondents are markedly higher than for those from other countries in this cluster. Conversely, we also confirm that conspiracy beliefs are higher among respondents in countries with a polarized political and media environment (H6b).

**TABLE 3:** One-way ANOVA for country of residence on conspiracy beliefs and misinformation beliefs.

Dependent variables	df	F	Sig.	Country	Mean score
Conspiracy beliefs	7	107.82	0.00	Philippines	5.83
				United States	5.19
				Hong Kong	5.03
				England	4.97
				Belgium	4.35
				Switzerland	4.31
				Canada	3.95
				New Zealand	3.86
Misinformation beliefs	7	172.63	0.00	Philippines	4.91
				Hong Kong	4.06
				United States	3.73
				England	3.51
				Switzerland	3.11
				New Zealand	3.05
				Canada	2.75
				Belgium	2.62

*Answer options for both misinformation and conspiracy beliefs ranged from 1 to 10, with the high end of the scale denoting high misinformation/conspiracy beliefs.*

With regards to sociodemographic differences, the results in Table 4 indicated that there were statistically significant differences in conspiracy beliefs by age and education, with mean scores indicating that younger age categories (18–34: *M* = 5.22; 35–54: *M* = 4.81) and lower educated individuals (*M* = 4.83) held higher conspiracy beliefs than older age categories (55 + : *M* = 3.99) and highly educated individuals (*M* = 4.53). As for misinformation beliefs, we again found that younger age categories (*M* = 4.03 for 18–34), lower educated individuals (*M* = 3.52), and women (*M* = 3.33) were more inclined to believe in misinformation than older age categories (*M* = 2.85 for 55 +), higher educated individuals (*M* = 3.36), and men (*M* = 3.56).

**TABLE 4:** One-way ANOVA for age, and independent samples *t*-test for gender and educational attainment, on conspiracy beliefs and misinformation beliefs.

Dependent variables	Independent variables	df	F	Sig.	Mean score
Conspiracy beliefs	**Age**	8,781	76.35	0.00	
	18–34				5.22
	35–54				4.81
	55 +				3.99
	**Gender**	8,781	13.82	0.24	
	Male				4.63
	Female				4.69
	**Education**	8,710	15.70	0.00	
	Secondary education or lower				4.83
	Tertiary education or higher				4.53
Misinformation beliefs	**Age**	8,781	91.62	0.00	
	18–34				4.03
	35–54				3.54
	55 +				2.85
	**Gender**	8,781	49.04	0.00	
	Male				3.56
	Female				3.33
	**Education**	8,710	4.23	0.00	
	Secondary education or lower				3.52
	Tertiary education or higher				3.36

*Answer options for both misinformation and conspiracy beliefs ranged from 1 to 10, with the high end of the scale denoting high misinformation/conspiracy beliefs.*

Subsequently, we present the (standardized) direct effects from the structural equation model (SEM). The model was estimated in SAS Version 9.4 using proc calis. Goodness-of-fit indices indicated that all eight models yielded a good fit to the data (RMSEA < 0.08, GFI > 0.90, CFI > 0.95, SRMR < 0.05). We included sociodemographic indicators in all models, but only present them in the full model in Supplementary Appendixes E,F. The associations of these indicators with conspiracy and misinformation beliefs were consistent in all regions.

**FIGURE 1:**
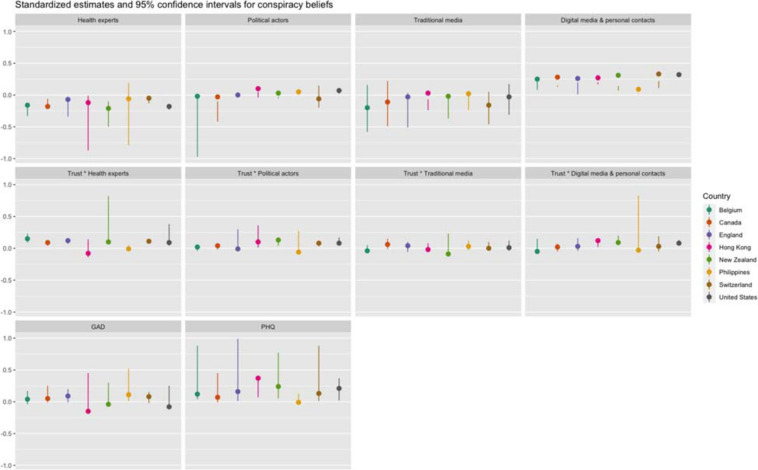
Direct standardized effects of predictors on conspiracy beliefs per country.

**FIGURE 2:**
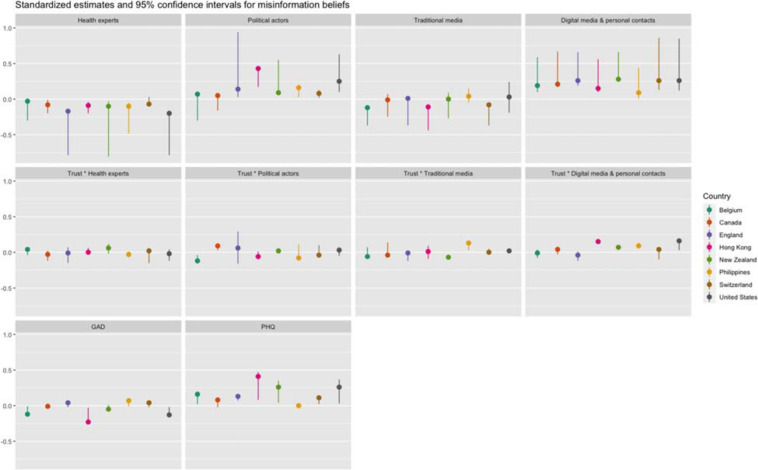
Direct standardized effects of predictors on misinformation beliefs per country.

Table 5 (see also Figures 1, 2) shows that conspiracy theory and misinformation beliefs were associated with exposure to several information sources about COVID-19—and the interactions with trust in these sources. In all countries except Switzerland, exposure to health experts was associated with lower conspiracy and misinformation beliefs. At the same time, exposure to political actors was associated with greater conspiracy beliefs in the U.S., Hong Kong, and the Philippines, and greater and misinformation beliefs in all countries/regions except Belgium and Canada. In terms of information from traditional media (television, radio, print news), analyses showed greater exposure was negatively associated with conspiracy beliefs and misinformation beliefs in Belgium and Switzerland only. In Canada, exposure to traditional media was associated with lower conspiracy beliefs, and in Hong Kong with lower misinformation beliefs only. Based on these results, we can partially confirm hypotheses 2a and 2b. Conversely, exposure to digital media and personal contacts was associated with greater conspiracy theory beliefs and misinformation beliefs in all countries/regions, confirming Hypotheses 1a and 1b.

**TABLE 5:** Direct standardized effects of predictors on conspiracy beliefs and misinformation beliefs per country.

	Belgium		Canada		England		Hong Kong	
	Conspiracy beliefs	Mis information beliefs	Conspiracy beliefs	Mis information beliefs	Conspiracy beliefs	Mis information beliefs	Conspiracy beliefs	Mis information beliefs
**Exposure to information**								
Health experts	−0.16**	−0.03	−0.18***	−0.08*	−0.07*	−0.17***	−0.12**	−0.09**
Political actors	−0.02	0.07	−0.03	0.05	0.00	0.14***	0.10**	0.43***
Traditional media	−0.20***	−0.12**	−0.11**	−0.01	−0.03	0.01	0.03	−0.11**
Digital media and personal contacts	0.25***	0.19***	0.28***	0.21***	0.26***	0.26***	0.27***	0.15***
**Interaction trust/exposure**								
Health experts	0.15***	0.04	0.09*	−0.03	0.12***	−0.01	−0.08*	0.00
Political actors	0.02	−0.12**	0.04	0.09**	−0.01	0.06	0.10**	−0.06
Traditional media	−0.04	−0.06	0.06*	−0.04	0.04	−0.01	−0.02	0.01
Digital media and personal contacts	−0.05	−0.01	0.02	0.04	0.03	−0.04	0.12***	0.15***
**GAD**	0.04	−0.12*	0.05	−0.01	0.09	0.04	−0.15**	−0.23***
**PHQ**	0.12*	0.16**	0.07	0.08	0.16**	0.13*	0.37***	0.41***

	**New Zealand**		**Philippines**		**Switzerland**		**United States**	
	**Conspiracy beliefs**	**Mis information beliefs**	**Conspiracy beliefs**	**Mis information beliefs**	**Conspiracy beliefs**	**Mis information beliefs**	**Conspiracy beliefs**	**Mis information beliefs**

**Exposure to information**								
Health experts	−0.21***	−0.10**	−0.06	−0.10*	−0.05	−0.07	−0.18***	−0.20***
Political actors	0.03	0.09*	0.05*	0.16***	−0.06	0.08*	0.07*	0.25***
Traditional media	−0.02	0.00	0.02	0.04	−0.16**	−0.08*	−0.03	0.03
Digital media and personal contacts	0.31***	0.28***	0.09*	0.09*	0.33***	0.26***	0.32***	0.26***
**Interaction trust/exposure**								
Health experts	0.10*	0.06	−0.01	−0.03	0.11**	0.02	0.09*	−0.02
Political actors	0.13**	0.02	−0.06	−0.08	0.08*	−0.04	0.08*	0.03
Traditional media	−0.09*	−0.07*	0.03	0.13**	−0.00	0.00	0.01	0.02
Digital media and personal contacts	0.09**	0.07*	−0.03	0.09*	0.03	0.04	0.08*	0.16***
**GAD**	−0.04	−0.05	0.11*	0.07	0.08	0.04	−0.08	−0.13*
**PHQ**	0.24***	0.26***	−0.01	0.00	0.13*	0.11*	0.21***	0.26***

*p < 0.05; **p < 0.01, ***p < 0.001. This analysis also includes sociodemographic indicators.

The association of exposure to information sources with conspiracy and misinformation beliefs was significantly moderated by trust in these sources in several instances, confirming hypothesis 3. Although results differed between countries, two main trends could be discerned. In several countries, we found that the effect of exposure to health actors differed by levels of trust: as trust in information from health actors increased, the negative association between exposure to health actors and conspiracy and/or misinformation beliefs became stronger. Furthermore, we also found that the effect of exposure to digital media was moderated by trust in these media: as trust in digital media increased, the (positive) association between exposure to digital media and conspiracy and/or misinformation beliefs increased as well. In short, information from health actors was more likely to be associated with lower conspiracy or misinformation beliefs for those who report high trust in these actors, while information from digital media was more likely to be associated with higher conspiracy or misinformation beliefs among those who report high trust in these media. While there are some additional significant effects of the interaction between exposure to and trust in information sources, there was no clear pattern among these across countries.

Anxiety was not associated with conspiracy or misinformation in most countries, although Hong Kong presents a clear exception. There, a higher score on the GAD was associated with lower conspiracy and misinformation beliefs. We also find similar associations in Belgium and the United States. However, feelings of depression were more strongly associated with conspiracy or misinformation beliefs across countries. In all countries/regions, except Canada and the Philippines, a higher score on the PHQ was associated with greater conspiracy and misinformation beliefs. The results of a robustness analysis of all countries combined (Supplementary Tables A6, A7) showed that these indicators also mediated the effect of exposure, confirming hypothesis 4 and hypothesis 5. Direct effects indicated that exposure to traditional media was strongly and negatively associated with both anxiety and depression, and that exposure to digital media and personal contacts was positively associated with anxiety and depression. Exposure to health experts was also positively associated with anxiety, while exposure to politicians was negatively associated with these feelings.

Finally, we considered the results of the control variables—which were included in all models. In terms of age, we found that older respondents held greater conspiracy beliefs, but lower misinformation beliefs than younger respondents, and that women held lower conspiracy and misinformation beliefs than men. No clear effects emerge in terms of educational attainment. These results were mostly in line with our earlier findings (see Table 4).

## Discussion

While the SARS-CoV-2 virus spread rapidly across the globe, many societies were also confronted with an inescapable spread of “viral” phenomena like misinformation and conspiracy theories. Conspiracy ideas and misinformation narratives are considered to be viral because the population dynamics underlying their spread hold many characteristic parallels to those involved in the spread of infections and communicable diseases: (1) they tend to spread at a higher pace through an ecosystem than other ideas, and (2) they might have serious consequences in terms of public health behavior and public safety (e.g., lower vaccination intentions Jolley and Douglas, 2014) and for political and macro-economic outcomes (decreased trust in governmental and health institutions Lutkenhaus et al., 2019).

The current study set out to investigate who believes in these “contagious” narratives and who does not. More specifically, we aimed to examine how exposure to communication channels is associated with beliefs in conspiracy theories and misinformation. Additionally, we tested the moderating role of one’s trust in these information and communication channels as well as the mediating role of depression and anxiety. Given the unprecedented global nature of the deeply mediatized COVID-19 pandemic, a cross-country comparison seemed to be the most appropriate method. Data collected in eight different countries across the globe at the height of the COVID-19 pandemic in late May 2020 provided interesting new insights.

The extent to which people believe in COVID-19 conspiracy theories and misinformation varies significantly across the various geographical regions as well as by socio-demographic characteristics. The Philippines, United States, and Hong Kong ranked as the top three for beliefs in conspiracy theories and misinformation. Significantly lower scores for both beliefs were found for Switzerland, Canada and New Zealand and Belgium. This finding suggests that citizens of specific countries in our dataset (Philippines, Hong Kong, and the U.S.) are more susceptible to these narratives while others (Canada, New Zealand, Switzerland, and Belgium) are more resilient. A potential explanation is the different political, media, and economic climates of the countries under scrutiny. Indeed, as recently theorized by Humprecht et al. (2020), a country’s resilience to misinformation and conspiracy theories depends on several political, media-systems related, and economic indicators such as the level of societal polarization in the nation and the amount of populist and partisan communication; the strength of public service media, and the overlap or fragmentation of news media audiences; and the adoption of social media. While a systematic, comparative analysis of these indicators on a global scale is lacking, it seems safe to claim that the Philippines (with the election of president Rodrigo Duterte Webb and Curato, 2019), Hong Kong [with its highly partisan media landscape and the on-going polarization around the question of independence (Wu and Shen, 2020)], and the United States [with the polarizing presidency of Donald Trump, the large advertising and social media markets, and the fragmented news media landscape (Mudde and Kaltwasser, 2018; Humprecht et al., 2020)] are indeed confronted with higher levels of populism and societal polarization and with weaker public service media systems compared to countries like Switzerland, Canada, and Belgium (e.g., Frissen et al., 2020).

In terms of socio-demographics, some interesting findings came to light. First, age was significantly associated with misinformation beliefs and conspiracy beliefs: younger respondents believed more strongly in these narratives than the older generations. This suggests that with age, one develops some type of resilience to misinformation. Second, gender was a significant factor for believing in misinformation but was not significant for conspiracies. Third, believing in conspiracies (but not misinformation) differed significantly across educational attainment: the higher the educational attainment, the weaker the belief in the COVID-19 conspiracy theories. While not significant, the opposite trend was found for misinformation beliefs. Although this corroborates previous findings (van Prooijen, 2017), our results indicate that misinformation and conspiracy theories are indeed similar, but substantially different, misinformation phenomena, particularly in terms of an individual’s susceptibility to these beliefs. It suggests that, in contrast to believing in conspiracy theories, misinformation beliefs are to a lesser extent a question of an individual’s level of education or news media literacy. In fact, highly educated people do not believe substantially less in misinformation narratives than lower educated people. Yet, the question of why this is the case remains still unanswered.

Beliefs in conspiracy theories and misinformation tend to be negatively associated with exposure to traditional media and positively associated with digital media and personal contacts. More specifically, exposure to COVID-19 related information through traditional news media sources such as newspapers, radio, and television, is associated with lower beliefs in conspiracy theories and misinformation narratives in Belgium and Switzerland. At the same time, exposure to digital media to acquire COVID-19 information is associated with greater conspiracy beliefs and misinformation in all countries/regions. With these results, we build on the findings of earlier studies that suggested that conspiracy thinking was rather associated with an avoidance of established and traditional media (Boberg et al., 2020), and that the digital media ecosystem rather than the traditional news media, is a hotbed for the development of mis- and disinformation beliefs (Shu et al., 2016; Vosoughi et al., 2018). In line with previous literature, we also found that exposure to health experts is associated with lower conspiracy beliefs (Humprecht et al., 2020). One would expect that more exposure to information from political actors would also decrease beliefs in conspiracies and misinformation, but surprisingly, results showed that this exposure is associated with greater conspiracy and misinformation beliefs in Hong Kong, the United States, and the Philippines, and not associated with these beliefs in most other countries/regions. This relationship may seem somewhat puzzling and provokes additional questions. Does this suggest that trust in politics functions in fact as a catalyst for beliefs in misinformation, which contrasts previous studies (e.g., Humprecht et al., 2020)? We do not think so. During these uncertain times, audiences depend on and trust politicians to convey accurate and up-to-date information so that they can make informed decisions regarding their personal health. However, insights about COVID-19 shift at a rapid pace, and information that is widely disseminated by media and politicians, is sometimes contradicted by the same actors a few days or weeks later due to new scientific insights into the virus (see worldwide discussions regarding the effectiveness of facemasks to decrease the odds of transmitting COVID-19) (Apuke and Omar, 2020; Pennycook et al., 2020). This ambiguity will result in higher appraisals of threat, stress or anxiety among audiences (Garfin et al., 2020). Such situations “may lead to the rapid generation of hypotheses, conjecture, and potentially CTs [conspiracy theories], particularly when the person is exposed to large volumes of information” (Georgiou et al., 2020, p. 2). This immediately explains some interaction effects we found as well—it is precisely those individuals who trust politicians most and are most exposed to them, that will feel the greatest need to believe in sometimes far-fetched theories to make sense of the ambiguous or contradictory information they regularly receive during the current crisis. The same goes for individuals with high exposure to and trust in digital media and personal contacts; they report greater misinformation beliefs. They consume a lot of (conflicting) information from information sources that they trust, which stimulates anxiety, stress, and fear. In order to make sense of this situation—and thus reduce anxiety—they generate or believe alternative explanations for this informational ambiguity. Important to note in this regard is that these cross-country results are likely driven by dynamics within a few countries in our study (e.g., the United States, Hong Kong, the Philippines—the same countries from which respondents reported the greatest conspiracy and misinformation beliefs).

Our data show that anxiety was not strongly associated with conspiracy beliefs or misinformation beliefs in most regions, while depression was associated with higher beliefs in both misinformation and conspiracy theories. Both indicators mediate the relationship between exposure to information sources and conspiracy/misinformation beliefs. This seems to be best interpreted by looking at the intersection between (coping with) stress, uncertainties, and threats on the one hand, and Beck’s cognitive theory of depression (Beck, 1967), on the other hand.

Even though we did not include stress as a measurement in the current study, previous studies have shown that stressful life events are a significant predictor for beliefs in conspiracy theories above and beyond other psychological distress factors such as anxiety (Swami et al., 2016). Nevertheless, we encourage future studies to look into the cognitive-theoretical approach more in detail in order to come to a better understanding of the association between depression and beliefs in misinformation and conspiracy theories.

## Limitations and Directions for Future Research

The findings of the present study are subject to some limitations. First and foremost, while we use data from eight different countries, all our data were cross-sectional. This means that none of the findings in the current study should be interpreted as causal but rather as correlational. Because there is no temporal ordering between data points, all arrows in the model follow merely theory-driven hypothesized paths. Recent examples of internationally comparative studies on the COVID-19 pandemic where the relationship between misinformation beliefs and anxiety and depression was reversed also exist (Généreux et al., 2020). We can only test causality and/or reciprocity if we use a multi-wave research design consisting of at least three time points (Ployhart and MacKenzie, 2015). That being said, we encourage future studies to investigate whether these associations follow the hypothesized directions by means of a longitudinal research design.

Second, while we collected data in eight different countries, it should be noted here that our cross-country comparison has also some limitations. At the moment of collecting the data (May 29, 2020–June 12, 2020 in all countries) several countries were in different stages of the pandemic. For example, whereas Hong Kong and several European countries already passed a first peak in terms of COVID-related deaths, cases in the United States were still surging. This means that our results should be interpreted with this in mind and may also provide potential explanations for (the lack of) some effects. Particularly in regions where the peak of the first COVID-19-wave had passed at the time of the study, media effects were smaller or absent, while they were more pronounced in regions in which the infection rate was still growing.

## Conclusion

While the world is fighting a pandemic, it is also fighting an infodemic (WHO, 2020b) in which falsehoods tend to spread faster, further, and more easily than truths. In reaction to this, people everywhere in the world have retrogressed back to their trusted, traditional news media channels as their main providers of pandemic-related information, but they have also become more inclined to believe conspiracy theories and misinformation. The latter is specifically the case when exposure to digital media and politicians is high, but less so when exposure to traditional media and health experts is high. Our comparative analysis of eight regions around the world suggests that this might be a result of the increasing occurrence of mis- and disinformation about the COVID-19 pandemic on digital media and the conflicting information that originates from politicians, while mainstream news media commonly attempt to “debunk” misinformation and conspiracy theories. Additionally, schemata and other cognitive processes that are associated with a sense of uncertainty and stress might set in motion a never-ending chain reaction in which people seek for more information to reduce uncertainty and stress, but in contrast stumble upon stress-evoking discourses.

## Data Availability Statement

The raw data supporting the conclusions of this article will be made available by the authors, without undue reservation.

## Ethics Statement

The studies involving human participants were reviewed and approved by the Research Ethics Board of the CIUSSS de l’Estrie—CHUS. The patients/participants provided their written informed consent to participate in this study.

## Author Contributions

DD, TF, KM, Ld’H, GL, OC-P, M-EC, MD, NP-C, SS, and MG were involved in the study conceptualization, design, and implementation. MG obtained the funding (principal investigator), and her coinvestigators. DD and TF were the study statisticians and were involved in the data analysis. DD, TF, KM, and Ld’H were involved in interpreting the data. DD, TF, KM, Ld’H, GL, and the CCOUC team wrote the first draft. All authors have read and agreed to the published version of the manuscript.

## Conflict of Interest

The authors declare that the research was conducted in the absence of any commercial or financial relationships that could be construed as a potential conflict of interest.

## Footnotes

1 There are several 5G-corona conspiracy theories circulating. Some advance the idea that the cellular network weakens the immune system and makes people therefore more susceptible to the virus. Others claim that the 5G masts are actively broadcasting the virus through the cellular infrastructure (see Vincent, 2020).
