# Supplementary material for: Beliefs in Conspiracy Theories and Misinformation About COVID-19: Comparative Perspectives on the Role of Anxiety, Depression and Exposure to and Trust in Information Sources
Source: Front Psychol. 2021 Apr 16;12:646394. doi: 10.3389/fpsyg.2021.646394 (PMC8085263; doi:10.3389/fpsyg.2021.646394)
Supplement: Supplementary file 1 [file Table_1.DOCX]

**Appendix A:** *Age and household comparison between country samples and population*

Table A1. Age distribution of sample and general population per country

|  | Category | Age distribution - population (%) | Weighted age distribution -sample (%) |
| --- | --- | --- | --- |
| Canada^1^ | 18-44 | 39.4 | 43.5 |
|  | 45-64 | 26.7 | 35.4 |
|  | 65+ | 18.0 | 21.1 |
| United States^2^ | 18-44 | 35.9 | 44.7 |
|  | 45-64 | 25.4 | 36.9 |
|  | 65+ | 16.5 | 18.4 |
| England^3^ | 18-44 | 37.9 | 44.9 |
|  | 45-64 | 25.6 | 32.4 |
|  | 65+ | 18.4 | 22.8 |
| Belgium^4^ | 18-44 | 35.6 | 40.4 |
|  | 45-64 | 26.8 | 37.6 |
|  | 65+ | 17.2 | 22.0 |
| Switzerland^5^ | 18-44 | 26.7 | 37.7 |
|  | 45-64 | 35.3 | 41.5 |
|  | 65+ | 17.8 | 20.8 |
| Hong Kong^6^ | 18-44 | 38.7 | 44.8 |
|  | 45-64 | 31.8 | 36.8 |
|  | 65+ | 17.9 | 18.4 |
| Philippines^7^ | 18-44 | 47.5 | 66.6 |
|  | 45-64 | 15.9 | 25.7 |
|  | 65+ | 4.7 | 7.7 |
| New Zealand^8^ | 18-44 | 39.9 | 46.9 |
|  | 45-64 | 24.9 | 33.2 |
|  | 65+ | 15.9 | 19.9 |

Note: 1. Statistique Canada, Estimations démographiques (septembre 2020). Adapté par l'Institut de la statistique du Québec. 2. United States census bureau (2020). National Population by Characteristics: 2010-2019. 3. Office for national statistics (2020). Estimates of the population for the UK, England and Wales, Scotland and Northern Ireland. 4. Directorate-General Statistics – Statistics Belgium (2020). Population by gender and age group in Belgium, 2010-2020. 5. Swiss confederation (2015). Switzerland's population 2014. 6. Demographic Statistics Section, Census and Statistics Department (2020). Table 002: Population by Age Group and Sex. 7. Philippine statistic authority (2019). Census based Population Projections using the results of the 2015 Census of Population. 8. Stats NZ (2020). National population estimates: At 30 June 2020.

Table A2. Household composition of sample and general population per country

|  | Category | Actual distribution - population^1^ (%) | Weighted distribution - sample (%) |
| --- | --- | --- | --- |
| Canada | Alone | 28.2 | 20.2 |
|  | Children | 52.3 | 27.3 |
|  | Other | 19.4 | 52.5 |
| United States | Alone | 27.9 | 21.9 |
|  | Children | 32.9 | 35.2 |
|  | Other | 39.2 | 42.9 |
| England | Alone | 30.6 | 20.7 |
|  | Children | 35.9 | 29.6 |
|  | Other | 33.6 | 49.7 |
| Belgium | Alone | 34 | 18.9 |
|  | Children | 36.1 | 27.5 |
|  | Other | 30.0 | 53.5 |
| Switzerland | Alone | 36 | 23.6 |
|  | Children | 32.9 | 26.4 |
|  | Other | 31.1 | 50.0 |
| Hong Kong | Alone | 18.3 | 6.5 |
|  | Children | 48.6 | 43.3 |
|  | Other | 33.2 | 50.2 |
| Philippines | Alone | 9.2 | 4.8 |
|  | Children | 50.1 | 44.4 |
|  | Other | 40.6 | 50.8 |
| New Zealand | Alone | 22.8 | 18.0 |
|  | Children | 34.8 | 34.9 |
|  | Other | 42.4 | 47.0 |

Note: 1. United nation departement of economic and social affairs (2019). Household size and composition. Retreived from <https://population.un.org/Household/index.html#/countries/756>

**Appendix B:** *Items measuring conspiracy theory beliefs*

To what extent do you agree with the following statements regarding the coronavirus crisis?

| 1. I believe that my government is hiding important information about the coronavirus. |
| --- |
| 2. I believe that the pharmaceutical industry is involved in the spread of the coronavirus. |
| 3. I believe that the medication already exists to prevent or treat the coronavirus. |
| 4. I believe the coronavirus was made intentionally in a laboratory. |
| 5. I believe the coronavirus was made by mistake in a laboratory. |
| 6. I believe there is a link between 5G technology and the coronavirus. |

Table A3. Internal consistency, standardized factor loadings, and correlations between items on conspiracy beliefs (varimax rotation) – three-item version

| (α = .77) | Factor loading | 1. | 2. | 3. | 4. | 5. | 6. |
| --- | --- | --- | --- | --- | --- | --- | --- |
| 1. | .74 | - |  |  |  |  |  |
| 2. | .88 | .46** | - |  |  |  |  |
| 3. | .87 | .44** | .68** | - |  |  |  |

Note. Answer options range from 1 (do not agree at all) to 10 (fully agree).

Table A4. Internal consistency, standardized factor loadings, and correlations between items on conspiracy beliefs (varimax rotation) – six-item version

| (α = .86) | Factor loading | 1. | 2. | 3. | 4. | 5. | 6. |
| --- | --- | --- | --- | --- | --- | --- | --- |
| 1. | .63 | - |  |  |  |  |  |
| 2. | .88 | .46** | - |  |  |  |  |
| 3. | .83 | .44** | .68** | - |  |  |  |
| 4. | .83 | .51** | .65** | .52** | - |  |  |
| 5. | .70 | .22** | .43** | .27** | .38** | - |  |
| 6. | .74 | .24** | .40** | .32** | .38** | .22** | - |

Note. Answer options range from 1 (do not agree at all) to 10 (fully agree).

**Appendix C:** *Items measuring misinformation beliefs*

To what extent do you agree with the following statements regarding the coronavirus crisis?

| 1. I believe the coronavirus is not transmitted in warm countries. |  |
| --- | --- |
| 2. I believe that the coronavirus will disappear after the outbreak. |  |
| 3. I believe that the sun or temperatures higher than 25C degrees prevent the coronavirus disease. | |
| 4. I believe that the new coronavirus can be transmitted through mosquito bites. |  |
| 5. I believe that spraying alcohol or chlorine all over my body will kill the new coronavirus. |  |

Table A5. Internal consistency, standardized factor loadings, and correlations between items on misinformation beliefs (varimax rotation)

| (α = .86) | Factor loading | 1. | 2. | 3. | 4. | 5. |
| --- | --- | --- | --- | --- | --- | --- |
| 1. | .85 | - |  |  |  |  |
| 2. | .77 | .60** | - |  |  |  |
| 3. | .82 | .65** | .55** | - |  |  |
| 4. | .74 | .54** | .42** | .48** | - |  |
| 5. | .80 | .57** | .50** | .56** | .57** | - |

Note. Answer options range from 1 (do not agree at all) to 10 (fully agree).

*
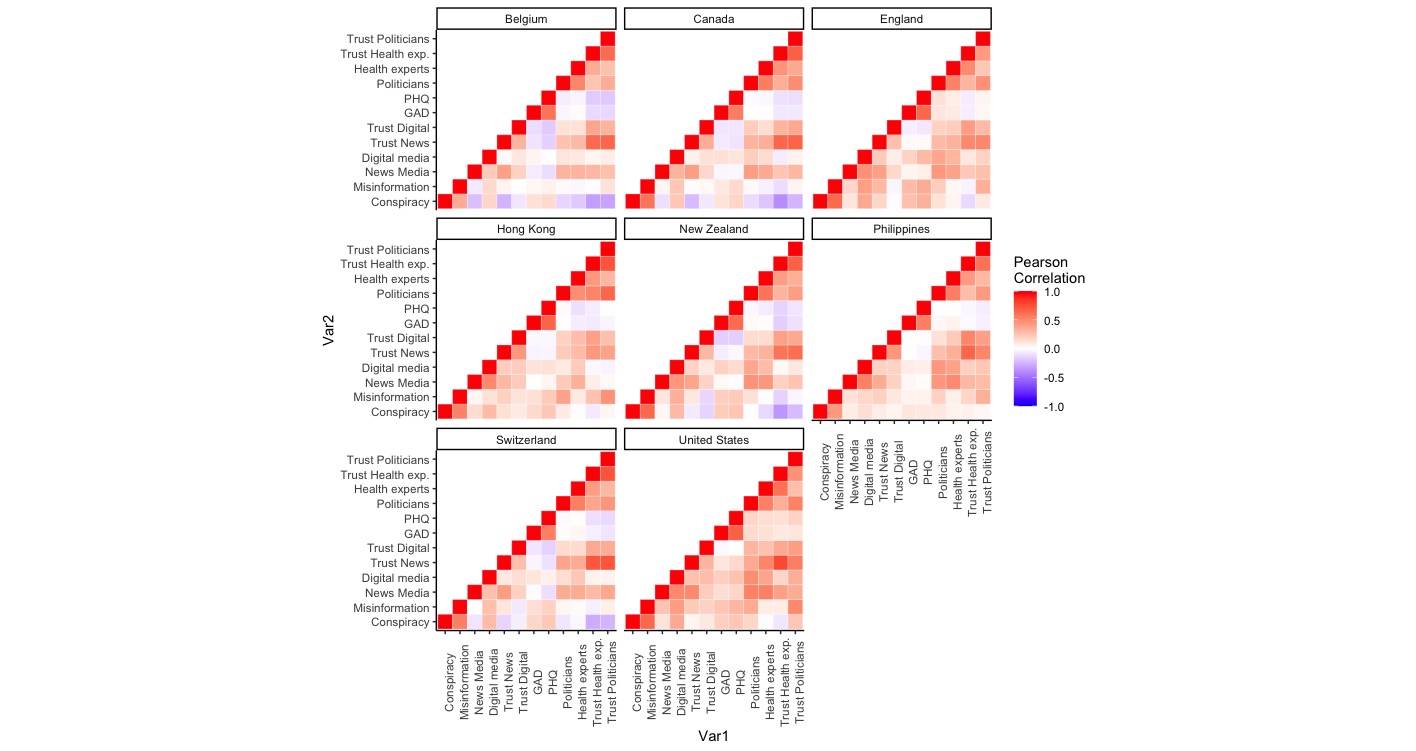
***Appendix D:** *Descriptive overview of conspiracy and misinformation beliefs, exposure to and trust in information sources, and anxiety and depression*

Figure A1. Heatmaps of correlation of conspiracy and misinformation beliefs, exposure to and trust in information sources, and anxiety and depression by country


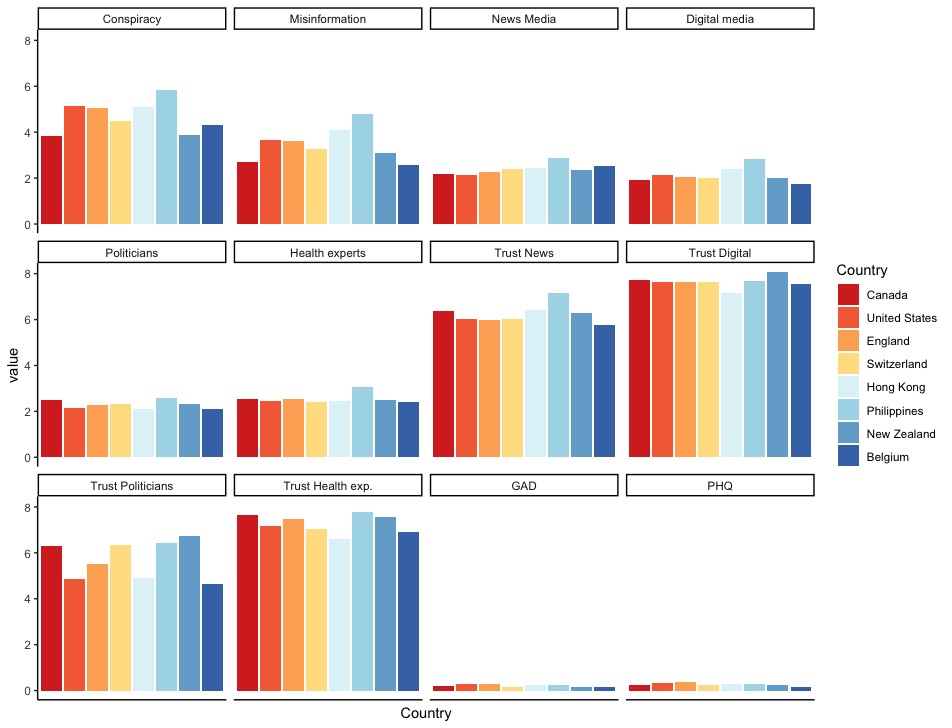


Figure A2. Bar plots of mean scores of conspiracy and misinformation beliefs, exposure to and trust in information sources, and standardized anxiety and depression scores by country

**Appendix E:** *Robustness check with all countries combined*

Table A6. Direct standardized effects of predictors on anxiety and depression, and on conspiracy beliefs (three-item) and misinformation beliefs

|  | GAD | | PHQ | | Conspiracy beliefs | | Misinformation beliefs |
| --- | --- | --- | --- | --- | --- | --- | --- |
| **Exposure to information sources** |  |  | |  | |  |  |
| Health experts | 0.15*** (.03) | | 0.01 (.03) | | -0.24*** (.04) | | -0.05 (.05) |
| Political actors | -0.07* (.03) | | 0.06 (.03) | | 0.19*** (.04) | | 0.21*** (.04) |
| Legacy media | -0.27*** (.02) | | -0.28*** (.02) | | -0.19*** (.03) | | -0.17*** (.03) |
| Digital media and personal contacts | 0.40*** (.02) | | 0.40*** (.02) | | 0.45*** (.03) | | 0.36*** (.03) |
| **Interaction trust and exposure** |  |  | |  | |  |  |
| Health experts |  | |  | | 0.06 (.01) | | -0.01 (.01) |
| Political actors |  | |  | | 0.04** (.01) | | -0.03* (.01) |
| Legacy media |  | |  | | 0.00 (.01) | | 0.01 (.01) |
| Digital media and personal contacts |  | |  | | 0.01 (.01) | | 0.05*** (.01) |
| **GAD** |  | |  | | 0.03 (.02) | | -0.05** (.02) |
| **PHQ** |  | |  | | 0.10*** (.02) | | 0.16*** (.02) |
| **Age** |  | |  | | 0.02 (.02) | | -0.02 (.01) |
| **Gender (ref: male)** |  | |  | |  | |  |
| Female |  | |  | | -0.01 (.01) | | -0.08*** (.01) |
| **Education (ref: secondary education or lower)** |  |  | |  | |  |  |
| Tertiary education or higher |  | |  | | 0.00 (.01) | | 0.01 (.01) |
| **Country of residence (ref: England)** |  |  | |  | |  |  |
| Belgium |  | |  | | 0.03 (.02) | | -0.02 (.02) |
| Canada |  | |  | | -0.17*** (.02) | | -0.13*** (.02) |
| Hong Kong |  | |  | | -0.04** (.02) | | 0.06*** (.02) |
| United States |  | |  | | 0.00 (.02) | | 0.03* (.01) |
| New Zealand |  | |  | | -0.12*** (.02) | | -0.04** (.01) |
| Switzerland |  | |  | | -0.03* (.02) | | -0.00 (.02) |
| Philippines |  | |  | | 0.05** (.02) | | 0.08*** (.02) |

Note: *: *p* < 0.05; **: *p* < 0.01, ***: *p* < 0.001.

Table A7. Indirect standardized effects of exposure to information sources on conspiracy beliefs (three-item) and misinformation beliefs, via anxiety and depression

|  | | Conspiracy beliefs | Misinformation beliefs |
| --- | --- | --- | --- |
| **Exposure to information sources** |  |  |  |
| Health experts | | 0.01 (.00) | -0.01 (.01) |
| Political actors | | 0.00 (.00) | 0.01** (.00) |
| Legacy media | | -0.04*** (.00) | -0.03*** (.00) |
| Digital media and personal contacts | | 0.05*** (.01) | 0.04*** (.01) |

Note: *: *p* < 0.05; **: *p* < 0.01, ***: *p* < 0.001.

**Appendix F:** *Robustness check using six-item conspiracy belief scale*

Table A8. Direct standardized effects of predictors on anxiety and depression, and on conspiracy beliefs (six-item) and misinformation beliefs

|  | GAD | | PHQ | Conspiracy beliefs | | Misinformation beliefs |
| --- | --- | --- | --- | --- | --- | --- |
| **Exposure to information sources** |  |  | |  |  |  |
| Health experts | 0.06 (.03) | 0.18*** (.03) | | -0.31*** (.04) | | -0.09 (.05) |
| Political actors | 0.02 (.03) | -0.08** (.03) | | 0.25*** (.04) | | 0.16** (.05) |
| Legacy media | -0.29*** (.02) | -0.28*** (.02) | | -0.17*** (.03) | | -0.11*** (.03) |
| Digital media and personal contacts | 0.40*** (.02) | 0.39*** (.02) | | 0.46*** (.03) | | 0.38*** (.03) |
| **Interaction trust and exposure** |  |  | |  |  |  |
| Health experts |  |  | | 0.06 (.01) | | -0.01 (.01) |
| Political actors |  |  | | 0.04** (.01) | | -0.03* (.01) |
| Legacy media |  |  | | 0.00 (.01) | | 0.01 (.01) |
| Digital media and personal contacts |  |  | | 0.01 (.01) | | 0.05*** (.01) |
| **GAD** |  |  | | 0.05** (.02) | | -0.03 (.020 |
| **PHQ** |  |  | | 0.08*** (.02) | | 0.13*** (.02) |
| **Age** |  |  | | 0.04* (.02) | | -0.04** (.02) |
| **Gender (ref: male)** |  |  | |  | |  |
| Female |  |  | | -0.01 (.01) | | -0.09*** (.01) |
| **Education (ref: secondary education or lower)** |  |  | |  |  |  |
| Tertiary education or higher |  |  | | -0.00 (.01) | | 0.00 (.01) |
| **Country of residence (ref: England)** |  |  | |  |  |  |
| Belgium |  |  | | 0.01 (.02) | | -0.03* (.02) |
| Canada |  |  | | -0.19*** (.02) | | -0.14*** (.02) |
| United States |  |  | | 0.01 (.02) | | 0.03 (.02) |
| New Zealand |  |  | | -0.10*** (.02) | | -0.05** (.02) |
| Switzerland |  |  | | -0.05** (.02) | | -0.01 (.02) |
| Philippines |  |  | | 0.07*** (.02) | | 0.08*** (.02) |

Note: *: *p* < 0.05; **: *p* < 0.01, ***: *p* < 0.001. Hong Kong was excluded from this analysis due to the fact that some items used to construct the six-item conspiracy beliefs scale were not presented here.

Table A9. Indirect standardized effects of exposure to information sources on conspiracy beliefs (six-item), via anxiety and depression

|  | | Conspiracy beliefs | Fake news beliefs |
| --- | --- | --- | --- |
| **Exposure to information sources** |  |  |  |
| Health experts | | 0.02** (.01) | 0.00 (.00) |
| Political actors | | -.00 (.01) | .00 (.00) |
| Legacy media | | -0.04*** (.01) | -0.03*** (.00) |
| Digital media and personal contacts | | 0.05*** (.01) | 0.04*** (.01) |

Note: *: *p* < 0.05; **: *p* < 0.01, ***: *p* < 0.001. Hong Kong was excluded from this analysis due to the fact that some items used to construct the six-item conspiracy beliefs scale were not presented here.
